# Canopy nitrogen distribution is optimized to prevent photoinhibition throughout the canopy during sun flecks

**DOI:** 10.1038/s41598-017-18766-0

**Published:** 2018-01-11

**Authors:** Mitsutoshi Kitao, Satoshi Kitaoka, Hisanori Harayama, Hiroyuki Tobita, Evgenios Agathokleous, Hajime Utsugi

**Affiliations:** 1 Hokkaido Research Center, Forestry and Forest Products Research Institute, Sapporo, 062-8516 Japan; 2 Department of Plant Ecology, Forestry and Forest Products Research Institute, Matsunosato 1, Tsukuba, 305-8687 Japan

## Abstract

As photoinhibition primarily reduces the photosynthetic light use efficiency at low light, sunfleck-induced photoinhibition might result in a fatal loss of carbon gain in the shade leaves within a canopy with barely positive carbon balance. We hypothesized that shade leaves at the lower canopy might retain a certain amount of leaf nitrogen (N_L_) to maintain energy consumption via electron transport, which contributes to circumventing photoinhibition during sunflecks to keep efficient utilization of low light during the rest period of daytime. We investigated excess energy production, a potential measure of susceptibility to photoinhibition, as a function of N_L_ distribution within a Japanese oak canopy. Optimal N_L_ distribution, which maximizes canopy carbon gain, may lead to a higher risk of photoinhibition in shade leaves during sunflecks. Conversely, uniform N_L_ distribution would cause a higher risk of photoinhibition in sun leaves under the direct sunlight. Actual N_L_ distribution equalized the risk of photoinhibition throughout the canopy indicated by the constant excess energy production at the highest light intensities that the leaves received. Such a homeostatic adjustment as a whole canopy concerning photoinhibition would be a key factor to explain why actual N_L_ distribution does not maximize canopy carbon gain.

## Introduction

Within a canopy, leaves acclimate to the growth light conditions, where sun-exposed leaves have a higher area-based leaf N pool (N_L_) and higher photosynthetic capacity than shaded leaves^1–4^. Such a gradient of leaf N along the canopy depth is considered as an efficient utilization of the limited N resource to maximize photosynthetic C gain over the canopy of an entire plant in response to the light gradient within a canopy^5–7^. However, the measured N_L_ distribution is more uniform than the theoretically optimal N_L_ distribution to maximize the canopy carbon gain^8–10^, which means that sun leaves show lower and shade leaves greater N_L_ than the optimal value^6,11^. Furthermore, Hikosaka^10^ reported that the optimal N_L_ distribution, considering both direct and diffuse light into account, was much steeper than that conventionally estimated under diffuse light only^6,7^. Several theoretical approaches have been conducted to explain the less steep declines in N_L_ than the light gradient within canopies accounting other constraints than light, such as leaf morphological plasticity^12^, photosynthetic capacity at the top of the canopy limited by the maximum nutrient concentration^13^, and hydraulic conductance^14^.

Recently, the importance of direct light, i.e., sunflecks within a canopy, was emphasized both in photosynthesis and photoinhibition. Sunflecks can significantly improve C gain in shade leaves of the lower canopy, while shade leaves may suffer from photoinhibition by the high photosynthetic photon flux density (PPFD) of sunflecks^15^. Photosynthetic efficiency is reduced during the recovery from photoinhibition on the transition from sunflecks to shade, which would result in a considerable reduction in plant productivity^16^. The degree of photoinhibition has been empirically linked to average or integrated light intensity in photosynthetic plant production models^17,18^. However, recent studies have reported that plants grown under fluctuating light environments upregulated the photoprotective mechanisms and electron transport compared with those grown under constant light conditions in response to sunflecks, even when the amounts of daily total irradiance are comparable^19,20^. This suggests that leaves may well acclimate to sun flecks (brief and strong irradiance) rather than averaged irradiance, so as to circumvent transient photoinhibition.

Photoinhibition primarily reduces the photosynthetic light use efficiency at low PPFD, i.e. a reduction of the initial slope of the photosynthetic light-response curve rather than the light-saturated photosynthetic rate^17^. In this context, sunfleck-induced photoinhibition may result in a fatal loss of carbon gain in the shade leaves with barely positive carbon balance^16^. To prevent photoinhibition, electron transport, mainly involved in photosynthesis and photorespiration^21^, plays an important role in consuming absorbed light energy as well as thermal energy dissipation^3,22,23^. Excess energy, neither consumed by electron transport nor dissipated as heat, is known to have a close relationship to photoinactivation of PSII under the inhibition of PSII repair^24,25^.

Leaf N participation for the circumvention of photoinhibition is specifically involved in electron transport, but not in xanthophyll-related thermal energy dissipation, since xanthophyll pigments, violaxanthin (C_40_H_56_O_4_), antheraxanthin (C_40_H_56_O_3_), and zeaxanthin (C_40_H_56_O_2_), do not contain any N^26^. As for the fractions of leaf N in Rubisco, and in proteins related to linear electron transport, there can be little differences in the fractions between upper and lower canopy leaves^27^. In this context, leaf N gradient within the canopy can closely reflect the gradient of photosynthesis-related proteins. The capacity of electron transport depends on N_L_, which is varied through intra-canopy light gradients^28,29^. Thus, shade leaves at the lower canopy may retain a certain amount of N_L_ to circumvent photoinhibition during sunflecks to keep efficient utilization of low PPFD during the rest period of daytime^16^.

We hypothesized that N_L_ should be distributed within a canopy to prevent photoinhibition both in shade leaves during sunflecks and sun leaves under the direct sunlight, leading to the less steep N_L_ distribution than the optimal N_L_ distribution. To test this hypothesis, we determined how excess energy production, which could be a possible measure of the susceptibility to photoinhibition^22,24,25,30^, was distributed within a canopy when N_L_ distribution was changed. We investigated photosynthetic traits in the leaves within a canopy of Japanese oak by gas exchange and chlorophyll fluorescence measurements, as well as light and N distribution within the canopy.

## Results

Total leaf area index (LAI), F_T_, in the forest was estimated to be 5.91 m^2^ m^−2^ based on the stratified clipping method conducted in 2001 and 2003. Based on the LAI cumulated from the canopy top (F), the light extinction coefficient of the canopy (K_L_) was 0.739 in Eq. 6. Based on the relationship between the relative light intensity (I/I_0_) and F (Eq. 6), we derived F of 15 leaves used for the photosynthetic measurements in 2007 by using relative light intensity at each leaf position. The actual distribution of N_L_ as a function of F in 2007 was revealed as follows:

$${{\rm{N}}}_{{\rm{L}}}=2.22\,\exp (-0.241{\rm{F}})+0.269({\rm{according}}\,{\rm{to}}\,{\rm{Eq}}{\rm{.}}\,7\,{\rm{in}}\,{\rm{Methods}}).$$

Based on the integration of the actual N_L_ distribution, total N per unit ground area (N_T_) was calculated as 8.59 g m^−2^. Optimal N distribution in the canopy was expressed as follows:

$${{\rm{N}}}_{{\rm{L}}}=5.24\,\exp (-0.739{\rm{F}})+0.269({\rm{according}}\,{\rm{to}}\,{\rm{Eq}}{\rm{.}}\,8\,{\rm{in}}\,{\rm{Methods}}).$$

N_L_ with uniform distribution was estimated to be 1.45 g m^−2^, estimated as N_T_/F_T_.

**Figure 1:**
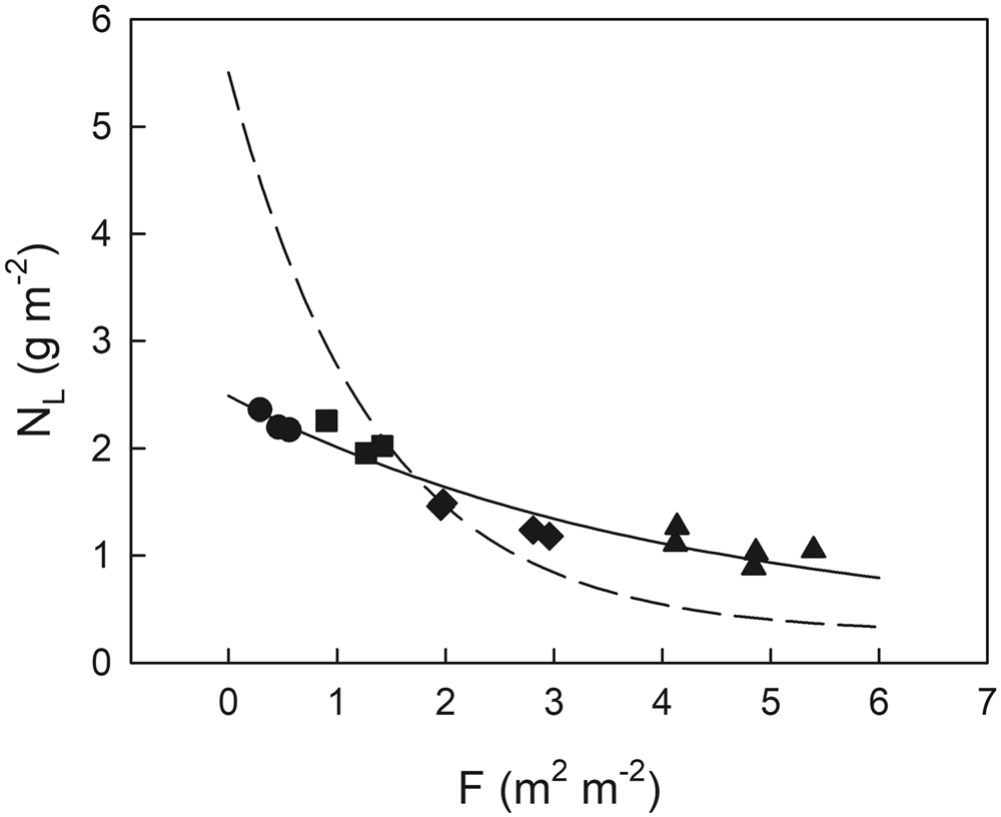
Actual leaf N (N_L_) distribution in the canopy of Japanese oak as a function of the leaf area index cumulated from the canopy top (F). Dashed line indicates optimal N_L_ distribution. Leaves were classified into four types based on their growth light environments (Q_int_): (1) deep shade (triangle, 0 < Q_int_ < 3 mol m^−2^ day^−1^), moderate shade (diamond, 3 < Q_int_ < 15 mol m^−2^ day^−1^), moderate sun (square, 15 < Q_int_ < 25 mol m^−2^ day^−1^), and typical sun leaves (circle, 25 < Q_int_ < 35 mol m^−2^ day^−1^).

Actual and optimal N_L_ distributions within the canopy are shown in Fig. 1. Optimal N_L_ was higher than the actual value in the case of F < 1.7 m^2^ m^−2^, whereas it was lower than the actual value in the case of F > 1.7 m^2^ m^−2^. F of 1.7 m^2^ m^−2^ corresponds to the average integrated daily quantum flux density in June (Q_int_) of 12 mol m^−2^ day^−1^ (Eq. 6).

**Figure 2:**
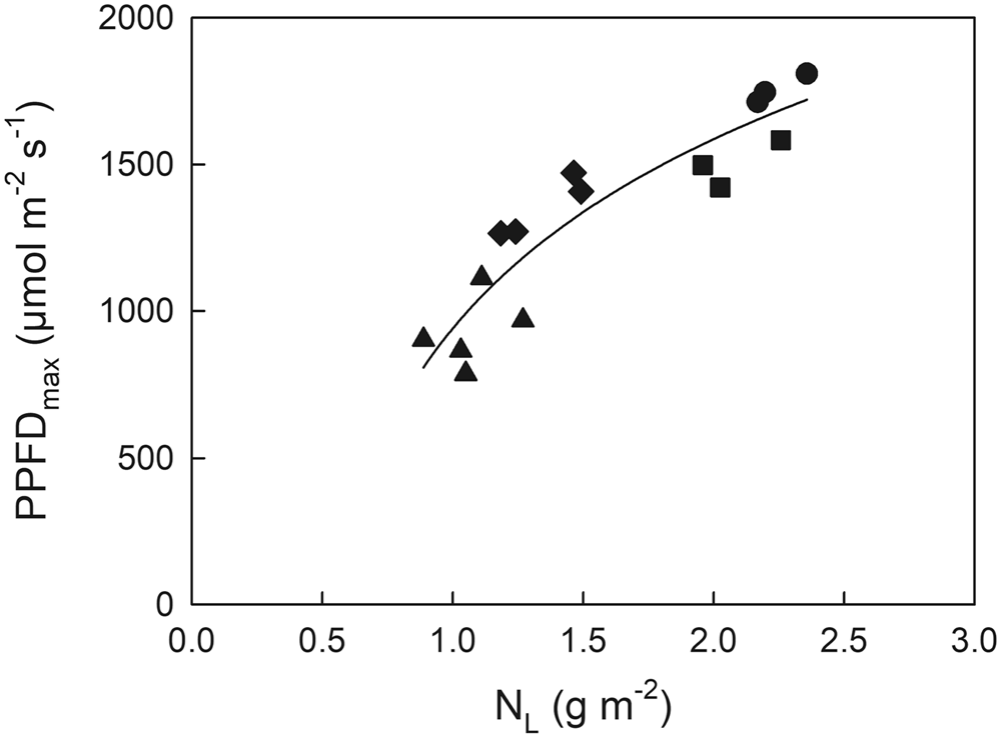
Maximal photosynthetic photon flux density (PPFD_max_) in June at various leaf positions with various leaf N pool (N_L_) within the canopy of Japanese oak. PPFD_max_ was estimated as average daily peak PPFD at each leaf position using the five highest values daily in June. Symbols are the same as in Fig. 1.

In the present study, we expediently classified the leaves into four types based on their growth light environments: (1) deep shade leaves (0 < Q_int_ < 3 mol m^−2^ day^−1^, n = 5); (2) moderate shade leaves (3 < Q_int_ < 15 mol m^−2^ day^−1^, n = 4); (3) moderate sun leaves (15 < Q_int_ < 25 mol m^−2^ day^−1^, n = 3); and (4) typical sun leaves (25 < Q_int_ < 35 mol m^−2^ day^−1^, n = 3). Hereafter, we often use “shade leaves” to refer to deep and moderate shade leaves and “sun leaves” to refer to moderate and typical sun leaves. Peak PPFD entering through the canopy (PPFD_max_), i.e. sunflecks, was considerably high (>700 µmol m^−2^ s^−1^), even for the deep shade leaves (Fig. 2).

**Figure 3:**
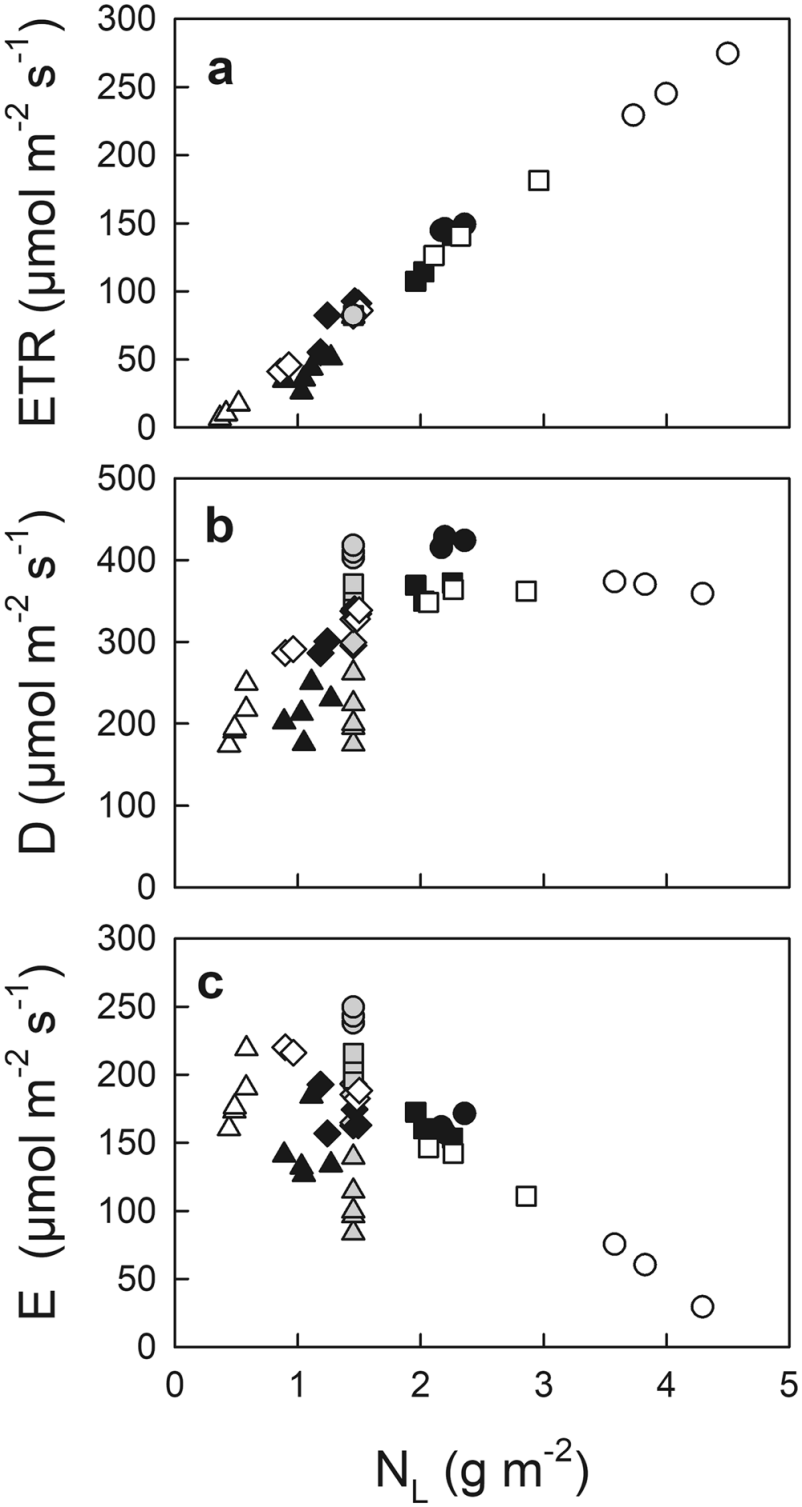
Electron transport rate (ETR) (**a**), thermal energy dissipation (D) (**b**), and excess energy (E) (**c**) at maximal photosynthetic photon flux density (PPFD_max_) as a function of leaf N pool (N_L_) with actual (closed), optimal distribution (open), and uniform distribution (grey symbols) in the canopy of Japanese oak. Symbols are the same as in Fig. 1.

**Figure 4:**
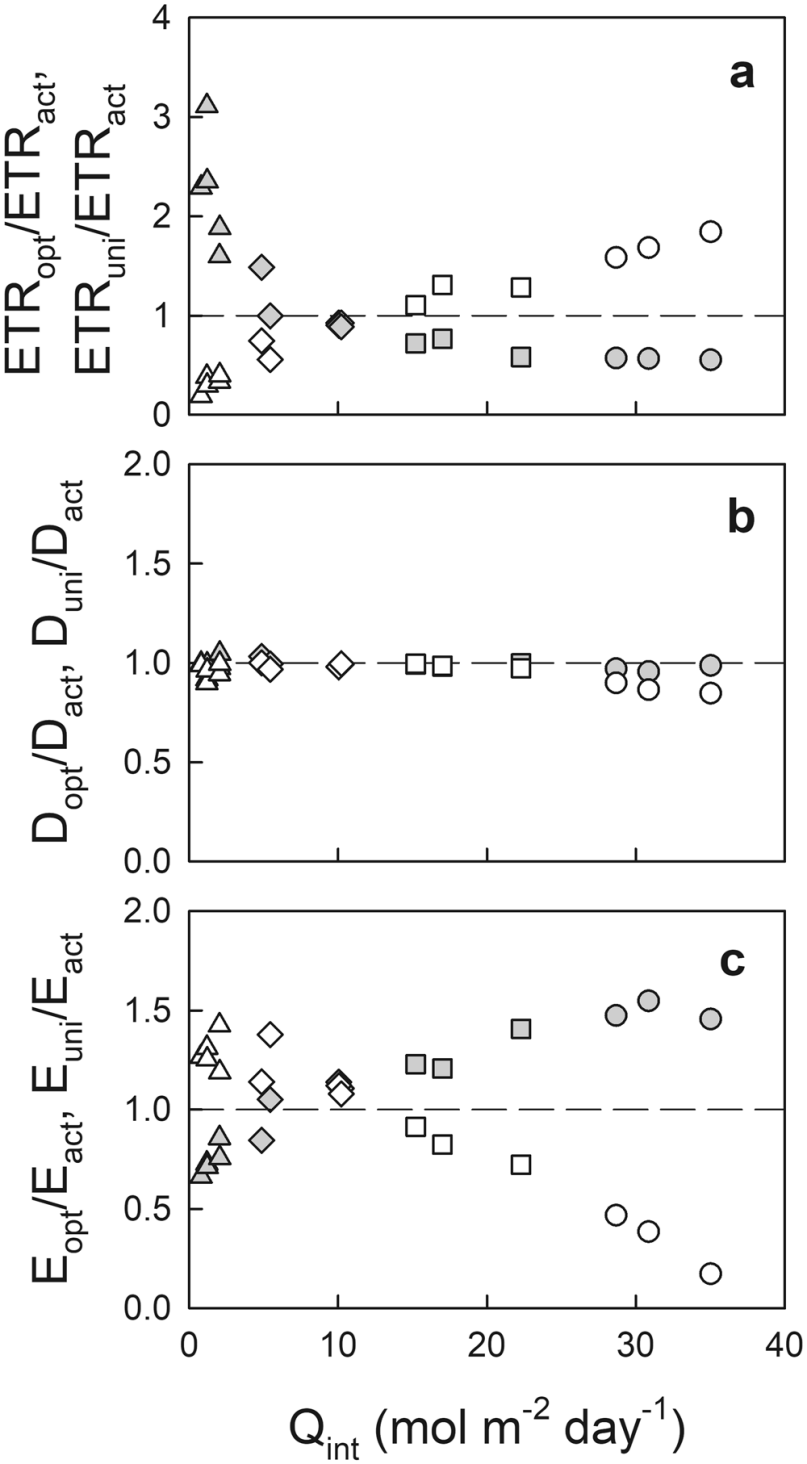
The ratio of ETR (**a**), D (**b**) and E (**c**) with optimal (open) and uniform N_L_ distribution (grey symbols) (ETR_opt_, D_opt_ and E_opt_; ETR_uni_, D_uni_ and E_uni_) to those with actual N_L_ distribution (ETR_act_, D_act_ and E_act_, respectively) at PPFD_max_ for a given leaf as a function the daily mean of the integrated photon flux density during leaf development (Q_int_). Dashed line indicates the ratios = 1. Symbols are the same as in Fig. 1.

Electron transport rate (ETR) with actual, optimal and uniform N_L_ distribution showed similar relationships as a function of N_L_ (Fig. 3a). Higher ETR in sun leaves but lower ETR in shade leaves was observed with optimal N_L_ distribution when compared with those with actual N_L_ distribution (Table 1). To investigate how ETR changed from the actual to the optimal and uniform N_L_ distribution, we showed ETR_opt_/ETR_act_ and ETR_uni_/ETR_act_ as a function of Q_int_ (Fig. 4a). Higher ETR_opt_/ETR_act_ was observed in leaves grown under higher Q_int_, compared among leaf types (Fig. 4a, Table 2). When compared with ETR in leaves with actual N_L_ distribution for each leaf type, relatively lower ETR in the deep shade leaves and relatively higher ETR in the typical sun leaves were observed with optimal N_L_ distribution (Table 2). Conversely, with uniform N_L_ distribution, higher ETR_uni_/ETR_act_ was observed in the deep shade leaves than the other leaves, where relatively higher ETR in the deep shade leaves and lower ETR in sun leaves were observed when compared with those in leaves with actual N_L_ distribution (Fig. 4a, Table 2).

**Table 1:** ETR, D and E (µmol m^−2^ s^−1^) in different leaf types of *Q*. *mongolica* at PPFD_max_ with actual, optimal and uniform N_L_ distribution.

Parameter	Leaf type				ANOVA
	Deep shade	Moderate shade	Moderate sun	Typical sun	
Actual N_L_					
ETR	38 ± 4^a^	80 ± 9^b^	121 ± 10^c^	147 ± 1^d^	F = 50.0 P < 0.001
D	214 ± 13^a^	315 ± 13^b^	364 ± 7^c^	423 ± 4^d^	F = 60.8 P < 0.001
E	143 ± 10	172 ± 8	162 ± 6	163 ± 4	F = 2.2 ns
Optimal N_L_					
ETR	12 ± 2^a^	64 ± 12^b^	149 ± 17^c^	250 ± 13^d^	F = 95.3 P < 0.001
D	205 ± 13^a^	311 ± 13^b^	358 ± 5^bc^	368 ± 4^c^	F = 44.5 P < 0.001
E	184 ± 10^c^	202 ± 10^c^	133 ± 11^b^	55 ± 14^a^	F = 32.9 P < 0.001
Uniform N_L_					
ETR	82 ± 0^a^	82 ± 0^b^	82 ± 0^b^	82 ± 0^b^	F = 19.7 P < 0.001
D	211 ± 15^a^	315 ± 10^b^	360 ± 7^c^	410 ± 4^c^	F = 51.3 P < 0.001
E	107 ± 9^a^	177 ± 8^b^	207 ± 5^c^	244 ± 3^d^	F = 54.3 P < 0.001

Values are means ± SE for each leaf type. Different letters indicate significant differences among the leaf types at P < 0.05, according to Holm’s pairwise comparisons. ns indicates non-significant effect of leaf type.

**Table 2:** The ratio of ETR, D and E with optimal and uniform N_L_ distribution (ETR_opt_, D_opt_ and E_opt_; ETR_uni_, D_uni_ and E_uni_) to those with actual N_L_ distribution (ETR_act_, D_act_ and E_act_, respectively) at PPFD_max_ in different leaf types of *Q*. *mongolica*. Values are means ± SE for each leaf type.

Parameter	Leaf type				ANOVA
	Deep shade	Moderate shade	Moderate sun	Typical sun	
Optimal N_L_					
ETR_opt_/ETR_act_	0.32 ± 0.04^a,*^	0.79 ± 0.09^b^	1.23 ± 0.06^c^	1.70 ± 0.08^d,*^	F = 81.3 P < 0.001
D_opt_/D_act_	0.96 ± 0.02^b^	0.99 ± 0.01^b^	0.99 ± 0.01^b^	0.87 ± 0.02^a,*^	F = 12.2 P < 0.001
E_opt_/E_act_	1.29 ± 0.04^c,*^	1.18 ± 0.07^c^	0.82 ± 0.06^b^	0.34 ± 0.09^a,*^	F = 47.6 P < 0.001
Uniform N_L_					
ETR_uni_/ETR_act_	2.25 ± 0.26^b,*^	1.07 ± 0.14^a^	0.69 ± 0.06^a,*^	0.56 ± 0.00^a,*^	F = 17.4 P < 0.001
D_uni_/D_act_	0.98 ± 0.02	1.00 ± 0.01	0.99 ± 0.00	0.97 ± 0.01	F = 0.54 ns
E_uni_/E_act_	0.74 ± 0.03^a,*^	1.04 ± 0.07^b^	1.28 ± 0.06^c,*^	1.49 ± 0.03^d,*^	F = 43.4 P < 0.001

Different letters indicate significant differences among the leaf types at P < 0.05, according to Holm’s pairwise comparisons. ns indicates non-significant effect of leaf type. *Indicates significant differences in ETR, D and E from those of actual N_L_ distribution in each leaf type at P < 0.05 with t-test.

Higher thermal energy dissipation (D) was generally observed in leaves grown under higher Q_int_ with any N_L_ distribution, whereas the slope become less steep in leaves with optimal N_L_ than the actual, and uniform N_L_ distribution (Fig. 3b, Table 1). With optimal N_L_ distribution, the typical sun leaves showed lower D_opt_/D_act_ than the other leaf types, and relatively lower D than that in the typical sun leaves with actual N_L_ distribution. Conversely, there was no significant differences in D_uni_/D_act_ among leaf types with uniform N_L_ distribution, and also there was no difference in D in leaves with uniform N_L_ distribution from that in leaves with actual N_L_ distribution within each leaf type (Fig. 4b, Table 2).

There was no difference in excess energy production (E) among leaf types with the actual N_L_ distribution (Fig. 3c, Table 1). A significantly higher E was observed in shade leaves than in sun leaves with optimal N_L_ distribution. Conversely, a significantly higher E was observed in sun leaves than in shade leaves with uniform N_L_ distribution (Fig. 3c, Table 1). Similarly, higher E_opt_/E_act_ was observed in shade leaves than in sun leaves with optimal N_L_ distribution, whereas higher E_uni_/E_act_ was observed in sun leaves than in shade leaves with uniform N_L_ distribution (Fig. 4c, Table 2). With optimal N_L_ distribution, deep shade leaves showed relatively higher E, but typical sun leaves showed relatively lower E compared with those in actual N_L_ distribution (indicated by the asterisks in Table 2). Conversely, with uniform N_L_ distribution, deep shade leaves showed relatively lower E, but sun leaves showed relatively higher E compared with those in actual N_L_ distribution.

**Figure 5:**
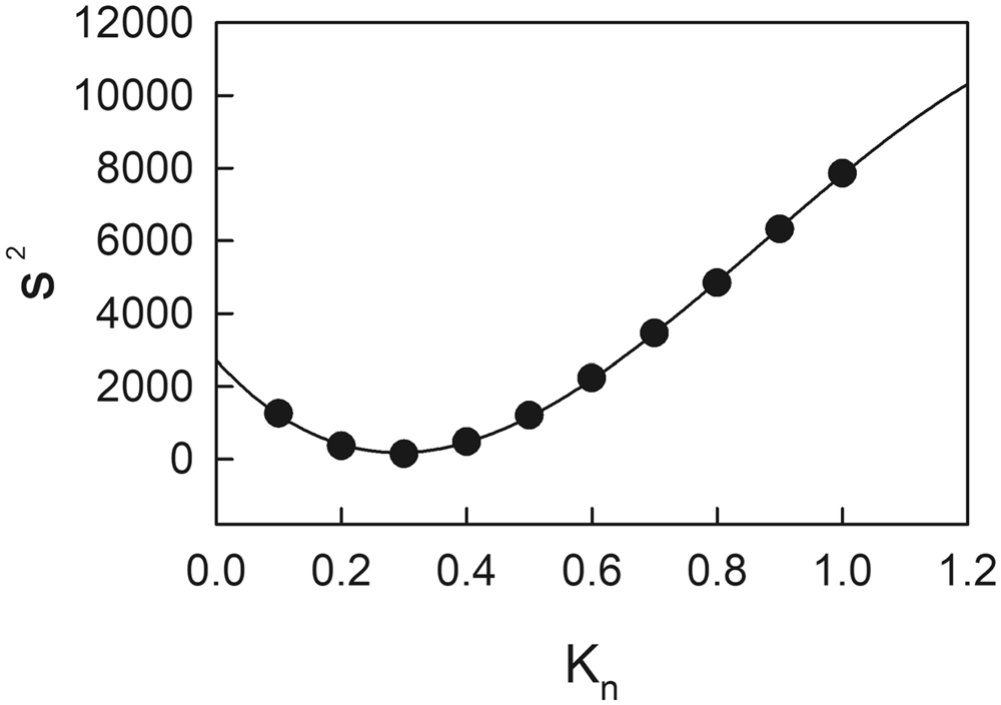
The relationship between the variance (s^2^) in excess energy production (E) at maximal photosynthetic photon flux density (PPFD_max_) and variable leaf N distribution coefficient (K_n_).

**Figure 6:**
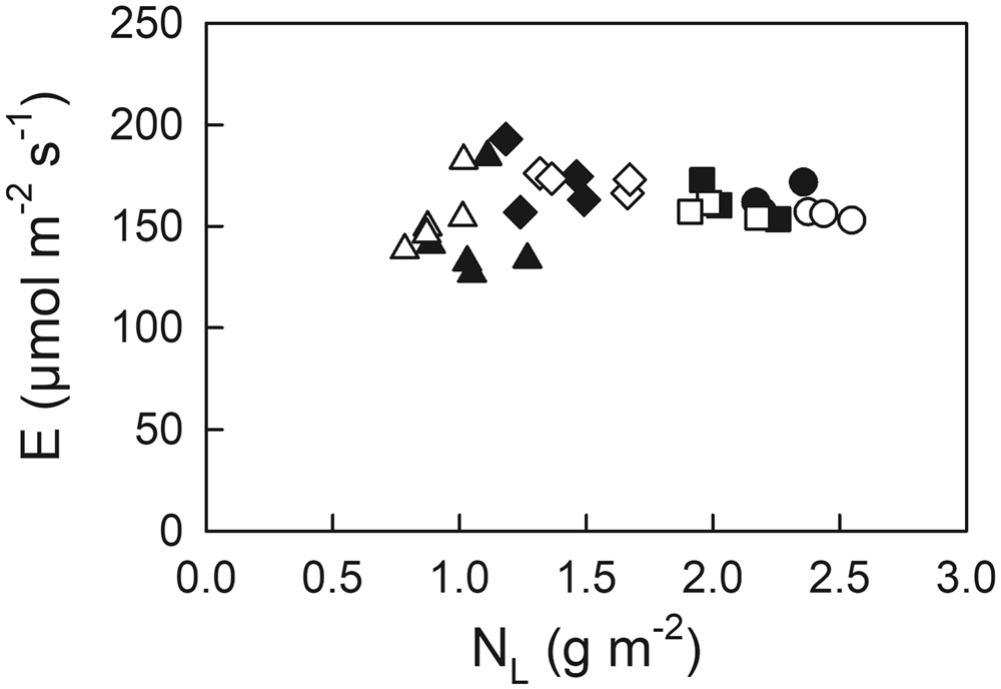
Excess energy production (E) at maximal photosynthetic photon flux density (PPFD_max_) as a function of leaf N pool (N_L_) with actual distribution (closed) and the distribution to minimize the variation in E (open symbols) in the canopy of Japanese oak. Symbols are the same as in Fig. 1.

Based on the relationship between variance in E (s^2^) and the leaf N distribution coefficient (K_n_), the K_n_ minimizing s^2^ was estimated as 0.291 (Fig. 5), which was very close to the K_n_ in the actual N_L_ distribution (0.241 in Eq. 7). We also found that the values of E at PPFD_max_ with the actual N_L_ distribution within the canopy of Japanese oak were very similar to those with the N_L_ distribution to maintain E constant, minimizing the variance in E (K_n_ = 0.291) (Fig. 6).

## Discussion

We investigated the fate of absorbed light energy (ETR, D, and E) in leaves with actual, optimal, and uniform N distribution within the canopy of Japanese oak. Excess energy is known to have a close relationship to photoinactivation of PSII under the inhibition of PSII repair^24,25^. There are two mechanisms involved in photoinhibition (defined as photoinactivation of PSII), i.e., an excess energy mechanism and a two-step mechanism^25^. As excess energy might be predominantly involved in photoinhibition in the field under visible light especially at the range of relatively high irradiance^25,30,31^, c.f. 700–1800 µmol m^−2^ s^−1^ during the sunflecks in the present study, we used the chlorophyll fluorescence parameter “excess energy,” as an empirical measure of the sensitivity of photoinhibitory damage based on the former mechanism. It is noteworthy that E during sunflecks for shade leaves or direct sunlight for sun leaves was stable among leaf types with the actual N_L_ distribution at various canopy depths, whereas a significantly higher E was observed in the shade leaves than sun leaves with the optimal N_L_ distribution; a significantly higher E in sun leaves than shade leaves with the uniform N_L_ distribution (Fig. 3c, Table 1). As photoprotective D was not enhanced in the leaves with decreased ETR (Table 2)^22^, the decreases in ETR in the shade leaves with an optimal N_L_ distribution, and in the sun leaves with a uniform N_L_ distribution both resulted in the increases in E. This also suggests that the photoprotective thermal energy dissipation would not respond enough to set off the changes in ETR with different N_L_ distributions as far as the present model prediction is concerned, partly because of the small variation in D in the fully acclimated leaves to their growth environments (Supplemental Fig. S1) as reported in long-term drought acclimated plants^32,33^.

In the present study, the optimal N distribution was estimated using the simple Beer’s law with an assumption that leaves received diffuse light only^6,7^. Hikosaka^10^ reported that the optimal N distribution under direct and diffuse light, a more realistic condition than diffuse light only^6,7^, was steeper than that under diffuse light only. However, from the viewpoint of photoinhibitory damage, the actual N distribution in the canopy of Japanese oak, which was less steep than the optimal distribution, might be inherently optimized to circumvent photoinhibitory damage at the whole-canopy level by maintaining excessive energy at a certain level under direct sunlight throughout the leaves grown at various light environments within a canopy.

We estimated canopy C gain based on light response curves built with different N distribution and incident PPFD with 1-min-intervals in July 2007 (details in Supplementary Information: Estimation of canopy C gain, Figs S2–S5). We used coefficients of light response curves measured at a leaf temperature of ≈27 °C, with no correction for the effects of air temperature, and air humidity on photosynthesis. Total canopy C gain was 24.9, 21.3, and 15.8 mol m^−2^ on the ground surface basis for optimal, even-E, which makes E constant throughout the canopy with K_n_ = 0.291 (Fig. 6), and uniform leaf N distribution, respectively. Even-E N distribution, showed a 15% decrease in canopy C gain integrated in July, during a photosynthetically most vigorous period, compared with optimal N distribution. Conversely, uniform N distribution resulted in a 36% decrease in canopy gain compared with the optimal N distribution. It is noteworthy, not only the sun leaves with higher leaf N_L_ with optimal canopy N distribution, but also the shade leaves with lower N_L_ compared with those with even-E N distribution contributed to the higher C gain as a consequence of reduced nighttime respiration rate (Supplementary Figure S5). In this estimation, a decrease in photosynthetic efficiency caused by photoinhibition under fluctuating light was not taken into account^16,19,20^. An enhanced recovery capacity from photoinhibition by bioengineering lead to an increase in biomass of tobacco plants by up to 20% in fluctuating light^16^. Thus, there is a possibility to reduce the difference in the canopy C gain between even-E and optimal N distribution, when the photoinhibitory effects on photosynthetic efficiency are accounted. In addition to an acclimation to short-term fluctuating light (sunflecks), as Japanese oak is a gap-dependent species, which needs a gap formation for its regeneration^2,23^, keeping relatively higher ETR in shade leaves than optimal would be a pre-conditioning for long-term fluctuating light (gap formation) for this species.

The cost of D1 protein turn-over, as a relevant process of PSII repair, is substantially low at most 0.5% of photosynthetic ATP production^34^, and the cost of relaxation of thermal energy dissipation is also reported to be substantially small, about 0.05% of the total photosynthetic electron flow under saturating light^26^. Therefore, the recovery cost from photoinhibition would be negligible for canopy C balance. Rather, reduced photosynthetic efficiency during the recovery from photoinhibition on transfer from sun flecks to shade may have a more significant adverse effect on canopy C gain^16^. In this context, circumvention of photoinhibition via an enhancement of ETR is essential to maintain the canopy C gain by preventing photoinhibition without any decrease in photosynthetic efficiency, in contrary to xanthophyll-related thermal energy dissipation.

Leaf photosynthetic capacity is closely related to leaf morphology such as leaf mass per area (LMA), where leaves with greater LMA generally have greater leaf area-based nitrogen contents and greater photosynthetic capacity^3,35,36^. During leaf maturation of Japanese oak, LMA reaches its maximum, accompanied with an increase in net photosynthetic rate, several weeks after the leaves are fully expanded and the light environment within a canopy is fixed (Tobita unpublished data^37^). Although the sun/shade anatomy of deciduous leaves is mainly determined by the light condition in the previous year^38^, less extent but some plasticity exists in leaf morphological change in response to current-year light environment^39^. For Japanese oak, LMA and net photosynthetic rate increased during June, after the leaf expansion had completed at the end of May (Tobita unpublished data). It was for this reason that we chose growth light environments (Q_int_, PPFD_max_) in June as determining factors for photosynthetic traits. The equalized amount of excessive energy production observed across the various light environments within a canopy (Figs 5 and 6) could be explained if the developments of photosynthesis (involved in ETR) and photoprotection (involved in D) along with the increase in LMA would be continued until the excessive energy production during sunflecks or direct sunlight drops below a certain level both in shade and sun leaves^19,20^.

The hypothesis of the present study: higher N_L_ than optimal is needed in the shade leaves to prevent photoinhibition during sunflecks, is partly similar to that proposed by Dewar *et al*.^12^, where a lower bound of LMA in the shade leaves exists, in relation to the limitation in leaf morphological plasticity^23^. Furthermore, the present study also proposes that the upper bound of N_L_ in sun leaves would be regulated as the minimum required to circumvent photoinhibition under direct sunlight, which might be related to the upper-bound constraint on photosynthetic capacity at the top of the canopy proposed by Lloyd *et al*.^13^ to explain the less steep N_L_ decline. In the present study, water stress during leaf development was considered to be negligible because of a considerable amount of spring snowmelt in the forest (mean maximum snow depth was 114 cm)^40^. Peltoniemi *et al*.^14^ proposed that limited hydraulic conductance for the sun leaves may result in a lower N_L_ in sun leaves with actual N_L_ distribution than the optimal one. In contrast, leaves acclimated to long-term drought often show higher N_L_ accompanied with higher ETR, than leaves grown without drought stress, to prevent photoinhibition under lower intercellular CO_2_ concentration by stomatal closure^32,33,41,42^. In this context, water stress on sun leaves induced by the limited hydraulic conductance in the upper canopy^43,44^ would not necessarily decrease N_L_ in sun leaves, but rather increase N_L_ for preventing photoinhibition. Further investigation is needed on such environmental stresses during leaf development influencing excess energy production to predict leaf N distribution within a canopy.

## Conclusion

The present study provides a novel insight into the canopy N_L_ distribution with respect to circumvention of photoinhibitory damage at the whole-canopy level like a homeostatic adjustment, where plants follow a strategy through which N_L_ distribution is not optimized for canopy C gain but rather regulated to prevent photoinhibition during sunflecks for shade leaves or direct sunlight for sun leaves. This regulation of N_L_ distribution contributes to keeping an efficient light utilization capacity during the daytime at the whole-canopy level, by means of minimizing a time lag in the recovery from photoinhibition^16^, which indicates an important biological plasticity in the response to light environment, and may suggest genetically-driven adjustments for successful acclimation in a changing light environment. Revealing such a physiological coordination of individual leaves for the benefit of the whole canopy will contribute to advancing the understanding of carbon–nitrogen interactions^45,46^ and energy flow in terrestrial ecosystems. Further studies with different species and at different environmental conditions are needed to demonstrate that the regulation of N_L_ distribution found in this study is an unequivocal biological mechanism occurring across species.

## Methods

### Study site

The present study was conducted in the experimental forest of the Hokkaido Research Center, Forestry and Forest Products Research Institute in Sapporo, Japan (43°N, 141°E; 180 m above sea level). Mean annual precipitation was approximately 900 mm and the mean annual temperature was 7.1 °C. The predominant species were Japanese white birch (*Betula platyphylla* Sukatchev var. *japonica* Hara) and Japanese oak (*Q*. *mongolica* Fisch. ex Ledeb. var. *crispula* (Blume) H. Ohashi), which comprised >75% of the plot basal area.

### Estimation of the photosynthetic photon flux density (PPFD) at various leaf positions within the canopy

Leaf morphological and physiological traits are determined by light environments under which leaves are developed^27^. We considered that light conditions during leaf development in June were important for leaves of *Q. mongolica* because photosynthesis reached its maximum during this period (Tobita unpublished data). A hemispherical photograph was taken by a digital camera (Coolpix 4500, Nikon, Tokyo, Japan) with a fisheye lens (Fisheye Lens, FC-E8, Nikon) right above each of the leaves that were used for the photosynthetic gas exchange and chlorophyll fluorescence measurements at the summertime in 2007. Based on the photographs, we estimated the incident PPFD for each leaf by using a canopy analysis software (WinScanopy, Regent Instruments Inc., Quebec, Canada)^47^ combined with the data of direct and diffuse radiation from the open sky measured by a pyrheliometer (CH1, Kipp & Zonen, Delft, The Netherlands) combined with an automatic solar tracker (ASTX-1, Prede, Tokyo, Japan) and a pyrheliometer (CM21, Kipp & Zonen) with 1-min intervals placed above the canopy in the experimental forest. Instantaneous incident PPFD at each leaf position was estimated as below:

$$\begin{array}{rcl}{\rm{PPFD}} & = & {\rm{ISF}}\times [{\rm{diffuse}}\,{\rm{radiation}}\,{\rm{from}}\,{\rm{the}}\,{\rm{open}}\,{\rm{sky}}]+[{\rm{direct}}\,{\rm{radiation}}]\\  &  & ({\rm{if}}\,{\rm{not}}\,{\rm{interrupted}}\,{\rm{by}}\,{\rm{above}}\,{\rm{leaves}}),\end{array}$$

or

1 $${\rm{PPFD}}={\rm{ISF}}\times [{\rm{diffuse}}\,{\rm{radiation}}\,{\rm{from}}\,{\rm{the}}\,{\rm{open}}\,{\rm{sky}}]({\rm{if}}\,{\rm{direct}}\,{\rm{radiation}}\,{\rm{was}}\,{\rm{interrupted}})$$

where ISF is the indirect (diffuse) site factor estimated by the WinScanopy software, penetration of the direct radiation is also assessed with the sun track simulated by the software. The average integrated daily quantum flux density at each leaf position in June (Q_int_; mol m^−2^ day^−1^) was calculated as a measure of growth light environment based on 1-min-interval PPFD from the 1st to 30th June, 2007. Average daily peak PPFD in June at each leaf position (PPFD_max_) was estimated using the daily five highest values during June^3^.

### Leaf area index (LAI), and light gradients within the canopy

We used the relationship between LAI and light gradients within the canopy of an inventory plot (50 × 50 m) of the experimental forest after Utsugi *et al*.^48^. The three predominant canopy tree species, *B. platyphylla*, *Q. mongolica*, and *Kalopanax septemlobus*, were used for the measurements of LAI during the summers of 2001 and 2003. Eight sample trees for each species (24 trees in total for each summer) were felled, and the gradient of LAI was determined by a stratified clipping method with 1-m strata intervals. PPFD within the canopy was also measured with PPFD sensors (Li-190SA, Li-Cor, NE, USA) with 1-m vertical intervals from 1 to 26 m along with permanent steel scaffolding within the canopy of *B. platyphylla*, *Q. mongolica*, and *K. septemlobus* in the area adjacent to the stratified clipping plot. The measurements were carried out under overcast conditions. Relative PPFD for each height was calculated as a mean of 200 replicates, with synchronized measurements of above and below canopy PPFD. The stand, consisting of *B. platyphylla*, *Q. mongolica* and *K. septemlobus*, was considered to be matured at that time since the age of the stand was about 90 years when the stratified clipping was conducted. We used the relationship between relative PPFD and LAI based on the pooled data for 2001 and 2003^48^ to estimate LAI for each leaf measured in 2007 from the relative PPFD. Relative PPFD at each leaf used for the photosynthetic measurement in 2007 was estimated as (Q_int_ at each leaf position)/(Q_int_ above the canopy), where Q_int_ above the canopy was 43.4 mol m^−2^ day^−1^ measured by the pyrheliometers placed above the canopy.

### Measurements of gas exchange and chlorophyll fluorescence

Measurements of gas exchange and chlorophyll fluorescence were conducted at the summertime of 2007 on mature leaves of a Japanese oak (approximately 23 m height and 95-years-old as of 2007), surrounded by the scaffolding described above with a portable photosynthesis measuring system (Li-6400, Li-Cor, Lincoln, NE, USA) combined with a leaf chamber fluorometer (Li-6400-40, Li-Cor). We selected a total of 15 leaves grown at various positions within the canopy (4 to 23 m in height). The net photosynthetic rate (A_n_), quantum yield of PSII electron transport (Φ_PSII_), photochemical efficiency of the open PSII (Fv′/Fm′) and excitation pressure adjusted for the efficiency of PSII photochemistry (1 − qP) Fv′/Fm′^24,30^ were measured at a photosynthetic steady state, where qP is photochemical quenching. The measurements were conducted under an ambient CO_2_ concentration of 360 µmol mol^−1^ and at various PPFD (0, 50, 100, 200, 300, 600, 1,000, 1,500, and 2,000 µmol m^−2^ s^−1^) provided by a red/blue LED array (Li-6400-40, Li-Cor), with blue light comprising 10% of the total PPFD. Leaf temperature was ≈27 °C during the measurements. After the measurements, the leaves were sampled and used for determination of N_L_ by the combustion method using an analysis system composed of a N/C determination unit (SUMIGRAPH, NC 800, Sumika Chem. Anal. Service, Osaka, Japan), a gas chromatograph (GC 8 A, Shimadzu, Kyoto, Japan), and a data processor (Chromatopac, C R6A, Shimadzu).

Electron transport rate (ETR) was calculated as ETR = Φ_PSII_ × leaf absorptance × light intensity × 0.5^22,49^. Leaf absorptance was calculated from a calibration curve between SPAD readings (measured with a SPAD chlorophyll meter, SPAD 502, Minolta, Osaka, Japan) and leaf absorptance^3^. The responses of electron transport to incident irradiance were fitted by the convexity equations^50^ as below:

2 $${{\rm{\theta }}\mathrm{ETR}}^{2}-({\rm{\varphi }}{\rm{PPFD}}+{{\rm{ETR}}}_{{\rm{\max }}})\,{\rm{ETR}}+{\rm{\varphi }}{\rm{PPFD}}\times {{\rm{ETR}}}_{{\rm{\max }}}=0$$

where ϕ is the initial slope (maximum quantum yield), θ the convexity of the curve, and ETR_max_ the maximum rate of electron transport.

The responses of Fv′/Fm′ and (1 − qP) Fv′/Fm′ to incident irradiance for each leaf were fitted as follows:

3 $${\rm{Fv}}^{\prime} /{\rm{Fm}}^{\prime} ={{\rm{y}}}_{0}+{\rm{a}}/(1+{({\rm{PPFD}}/{{\rm{x}}}_{0})}^{{\rm{b}}})$$

4 $$(1-{\rm{qP}}){\rm{Fv}}^{\prime} /{\rm{Fm}}^{\prime} ={\rm{a}}(1-\exp (-{\rm{bPPFD}}))$$

Analogous to ETR, thermal dissipation (D) and excess energy production (E) were estimated from (1 − Fv′/Fm′) × leaf absorptance × light intensity × 0.5 and ((1 − qP) Fv′/Fm′) × leaf absorptance × light intensity × 0.5, respectively^22,24^. Each coefficient in Eqs 2–4 was fitted as a function of N_F_ defined as (N_L_ – N_b_) of the leaves used for the chlorophyll fluorescence measurements, where N_b_ is the x-intercept of the maximum rate of photosynthesis (A_max_) − N_L_ relationship, which can be regarded as non-photosynthetic leaf structural N content. Conversely, N_F_ can be regarded as photosynthetically-functional leaf N content^6,51^. A_max_ was estimated from the light response of A_n_ based on the convexity equations^50^, as below:

5 $$\theta {({{\rm{A}}}_{{\rm{n}}}+{{\rm{R}}}_{{\rm{d}}})}^{2}-(\varphi {\rm{P}}{\rm{P}}{\rm{F}}{\rm{D}}+{{\rm{A}}}_{max})({{\rm{A}}}_{{\rm{n}}}+{{\rm{R}}}_{{\rm{d}}})+\varphi {\rm{P}}{\rm{P}}{\rm{F}}{\rm{D}}\,\ast \,{{\rm{A}}}_{max}=0$$

where R_d_ is the dark respiration rate, ϕ the initial slope (maximum quantum yield), and θ is the convexity of the curve. The relationships between N_F_ and photosynthesis-related coefficients are summarized in Supplemental Fig. S1. Based on the coefficients of light responses in ETR, Fv′/Fm′, and (1 − qP) Fv′/Fm′ as a function of N_F_, we extrapolated the light responses in leaves that have various N_L_ content with optimal and uniform N_L_ distribution.

### Actual, optimal and uniform leaf N distribution

We derived the light extinction coefficient of the canopy, K_L_^52^ as follows:

6 $${\rm{I}}/{{\rm{I}}}_{0}=\exp (-{{\rm{K}}}_{{\rm{L}}}{\rm{F}})$$

where F is the LAI cumulated from the canopy top, and I and I_0_ are PPFD at F and the canopy top, respectively. Actual N_L_ distribution at depth F in the canopy is also described by an exponential function of F with a photosynthetic leaf N distribution coefficient (K_n_):

7 $${{\rm{N}}}_{{\rm{L}}}=({{\rm{N}}}_{0}\, - \,{{\rm{N}}}_{{\rm{b}}})\exp (-{{\rm{K}}}_{{\rm{n}}}{\rm{F}})+{{\rm{N}}}_{{\rm{b}}}$$

where N_0_ is the N_L_ of the leaves at the canopy top. Optimal N_L_ distribution was derived after Anten *et al*.^6^ as follows:

8 $${{\rm{N}}}_{{\rm{L}}}={{\rm{K}}}_{{\rm{L}}}{{\rm{N}}}_{{\rm{TF}}}/(1-\exp (-{{\rm{K}}}_{{\rm{L}}}{{\rm{F}}}_{{\rm{T}}}))\exp (-{{\rm{K}}}_{{\rm{L}}}{\rm{F}})+{{\rm{N}}}_{{\rm{b}}}$$

With

9 $${{\rm{N}}}_{{\rm{T}}{\rm{F}}}={{\rm{N}}}_{{\rm{T}}}\, - \,{{\rm{N}}}_{{\rm{b}}}{{\rm{F}}}_{{\rm{T}}}$$

where N_TF_ is the total free nitrogen in the canopy and N_T_ and F_T_ are the total leaf N pool and LAI per unit ground area, respectively. N_T_ is calculated based on the integration of Eq. 2 ^11^. A N_L_ distribution is defined as uniform when all leaves have the same N_L_ irrespective of leaf position. Therefore, N_L_ with uniform distribution is determined as:

10 $${{\rm{N}}}_{{\rm{L}}}={{\rm{N}}}_{{\rm{T}}}/{{\rm{F}}}_{{\rm{T}}}$$

Based on the estimated N_L_ with optimal and uniform distribution, we calculated ETR, D, and E under the maximum PPFD during sunflecks for each leaf by using the N_L_-dependent photosynthetic coefficients (Supplemental Fig. S1) and Eqs 2–4.

We hypothesized that canopy N might be distributed to maintain E constant throughout the canopy during sunflecks, thus to keep an efficient light utilization capacity by preventing photoinhibition for all leaves across sun and shade environments. Therefore, we estimated the leaf N distribution coefficient (K_n_) that minimized the variance in E at PPFD_max_ across the different leaf types within the canopy, using Eq. 8 with various K_n_ (0.1 to 1.0 with an interval of 0.1) instead of K_L_ as follows:

11 $${{\rm{N}}}_{{\rm{L}}}={{\rm{K}}}_{{\rm{L}}}{{\rm{N}}}_{{\rm{TF}}}/(1-\exp (-{{\rm{K}}}_{{\rm{n}}}{{\rm{F}}}_{{\rm{T}}}))\exp (-{{\rm{K}}}_{{\rm{n}}}{{\rm{F}})+{\rm{N}}}_{{\rm{b}}}$$

Variance (s^2^) in E was estimated as follows:

12 $${{\rm{s}}}^{2}=\frac{1}{{\rm{n}}}\sum _{i\,=1}^{{\rm{n}}}{({{\rm{E}}}_{{\rm{i}}}-\bar{{\rm{E}}})}^{2}$$

### Statistical analysis

We expediently classified the leaves into four types based on their growth light environments: (1) deep shade leaves (0 < Q_int_ < 3 mol m^−2^ day^−1^, n = 5); (2) moderate shade leaves (3 < Q_int_ < 15 mol m^−2^ day^−1^, n = 4); (3) moderate sun leaves (15 < Q_int_ < 25 mol m^−2^ day^−1^, n = 3); and (4) typical sun leaves (25 < Q_int_ < 35 mol m^−2^ day^−1^, n = 3). One-way analysis of variance (ANOVA) was used to test the differences among leaf types in ETR, D, and E with actual, optimal, or uniform N_L_ distribution, and the ratio of ETR, D, and E with optimal or uniform to actual N_L_ distribution^53^. Furthermore, when the ANOVA returned an overall significant effect of leaf type, the means were further tested using Holm’s pairwise comparisons. The threshold for statistical significance was predefined at an alpha level of 0.05.

### Data availability

All data used in this manuscript are present in the manuscript and its supplementary information.

## Electronic supplementary material

Supplementary Information
