# Supplementary material for: Canopy nitrogen distribution is optimized to prevent photoinhibition throughout the canopy during sun flecks
Source: Sci Rep. 2018 Jan 11;8:503. doi: 10.1038/s41598-017-18766-0 (PMC5764975; doi:10.1038/s41598-017-18766-0)
Supplement: Supplementary file 1 — Supplementary Information [file 41598_2017_18766_MOESM1_ESM.pdf]

Canopy nitrogen distribution is optimized to prevent photoinhibition throughout the canopy during sun flecks

Mitsutoshi Kitao, Satoshi Kitaoka, Hisanori Harayama, Hiroyuki Tobita, Evgenios Agathokleous, Hajime Utsugi

Supplementary Figure S1

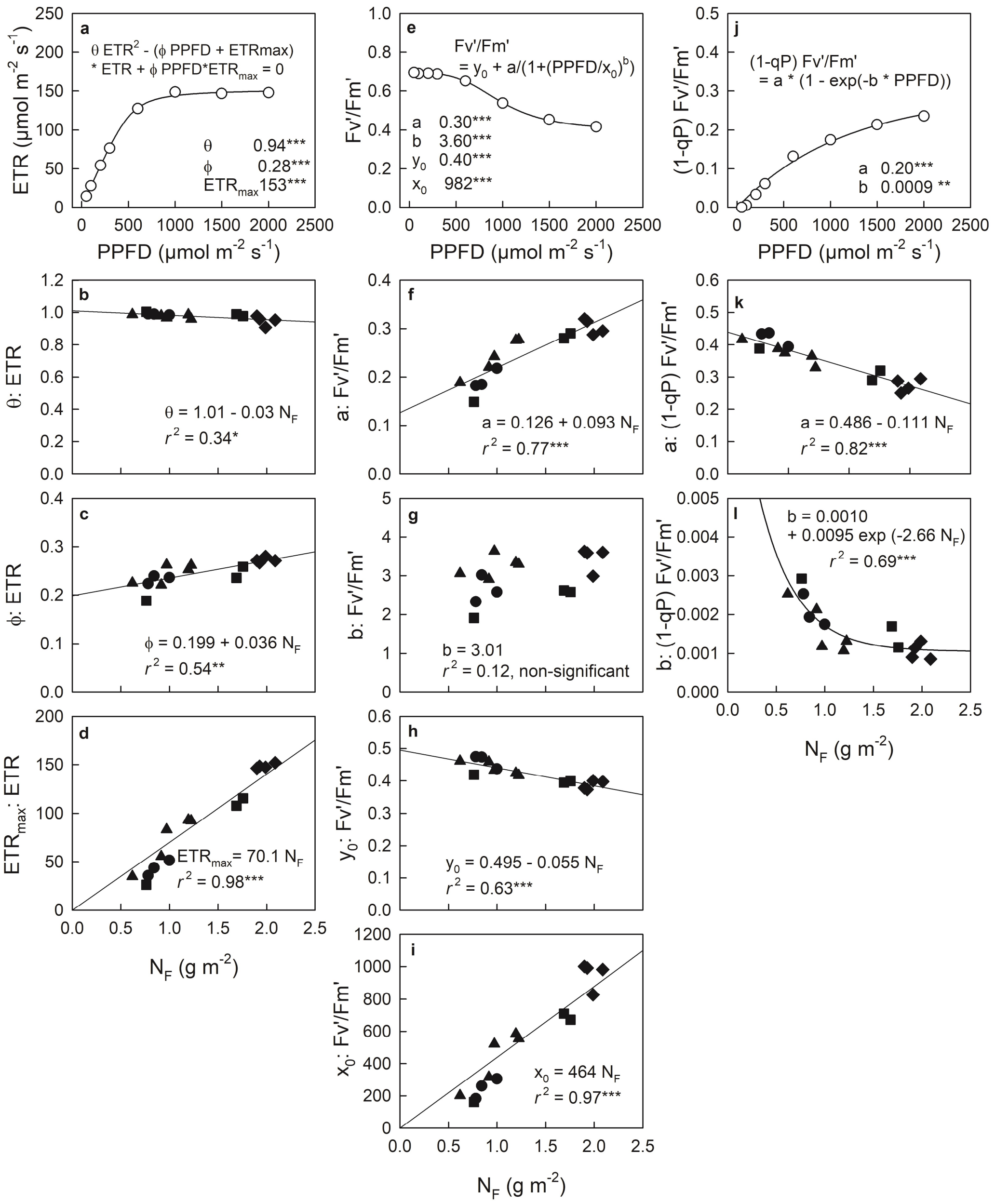

Supplementary Figure S1. Representative light-response curves of electron transport rate (ETR) (a), Fv′/Fm′ (e), and (1-qP) Fv′/Fm′ (j) in a typical sun leaf (Q_{int} = 33.6 mol m^{-2} day^{-1}) of Japanese oak. Curve fittings were conducted as described in equations in the section Materials and Methods. Coefficients of the equations of ETR (b to d), Fv′/Fm′ (f to i), and (1 − qP) Fv′/Fm′ (k and l) were regressed as a function of N_F. Leaves were classified into four types based on their growth light environments (Q_{int}): 1) deep shade (triangle, 0 < Q_{int} < 3 mol m^{-2} day^{-1}), moderate shade (diamond, 3 < Q_{int} < 15 mol m^{-2} day^{-1}), moderate sun (square, 15 < Q_{int} < 25 mol m^{-2} day^{-1}), and typical sun leaves (circle, 25 < Q_{int} < 35 mol m^{-2} day^{-1}). *denotes significance at P < 0.05, **P < 0.01, and ***P < 0.001.

Estimation of canopy C gain

We estimated canopy C gain with different leaf N (N_L) distribution; photosynthetically optimal: making canopy C gain maximum, even-E (excess energy): making E constant throughout the canopy, and uniform N distribution. The response of leaf-area-based net photosynthetic rate (A_n) to incident irradiance was fitted by the convexity equation as below:

$$\theta(A_n + R_d)^2 - (\phi PPFD + A_{max})(A_n + R_d) + \phi PPFD*A_{max} = 0 ,$$

where R_d is the dark respiration rate, ϕ is the initial slope (maximum quantum yield), θ is the convexity of the curve, and A_{max} is the maximum rate of photosynthesis. We determined the coefficients of the equations for totally 15 leaves grown within the canopy of Japanese oak. Based on the relationships between the coefficients and photosynthetically functional leaf N (N_F), we derived light response curve for each leaf with different N_L distribution (Supplementary Figure S2).

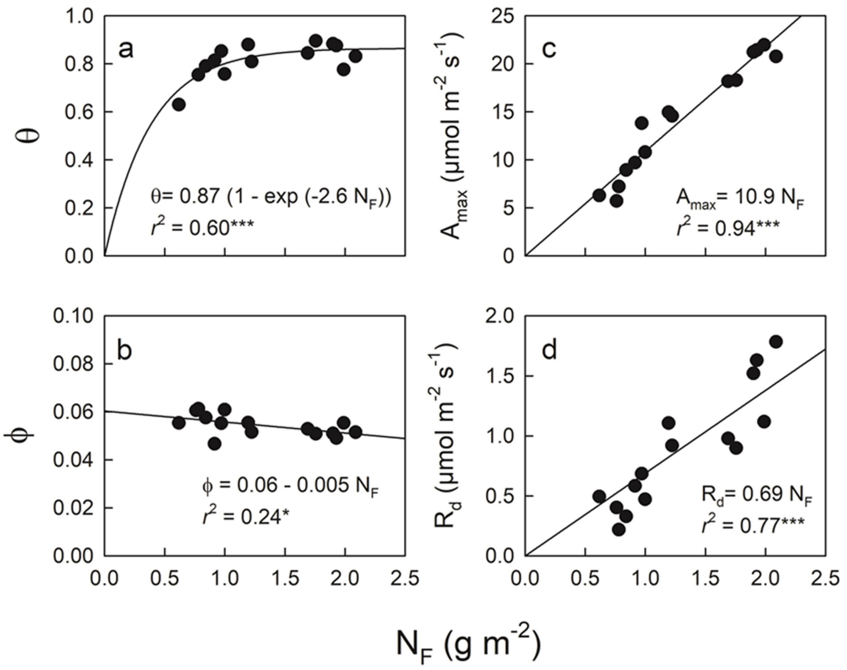

Supplementary Figure S2. Coefficients of light response curve (θ, ϕ, A_{max}, and R_d) as a function of N_F with actual leaf N distribution within the Japanese oak canopy.

We estimated 1-min-interval incident PPFD for each leaf during July 2007, based on the hemispheric photographs and the data of direct and diffuse radiation from the open

sky (details are described in Methods in the main text). Although other environmental factors such as air temperature and relative humidity are not taken into account, based on the 1-min-interval PPFD and leaf-N-dependent light response curve, we derived the relationship between integrated leaf-area-based C gain and LAI cumulated from the canopy top (F) for each leaf (Supplementary Figure S3).

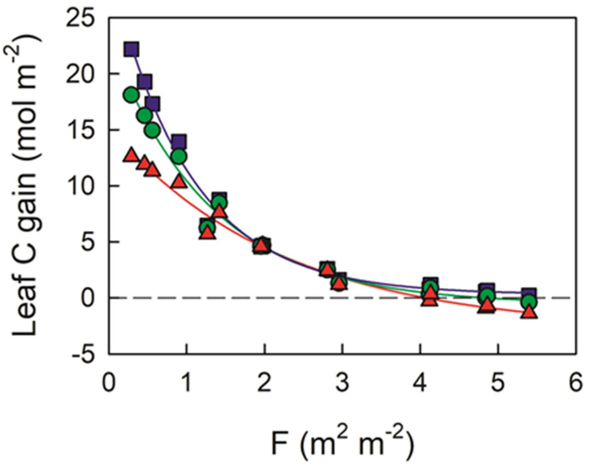

Supplementary Figure S3. Integrated leaf C gain of Japanese oak during July as a function of F with optimal (blue square), even-E (green circle), and uniform (red triangle) N_L distributions.

Canopy C gain was estimated as an integration of [LAI] x [leaf-area-based C gain] for each stratum with 1-m-intervals throughout the canopy (Supplementary Figure S3 & S4).

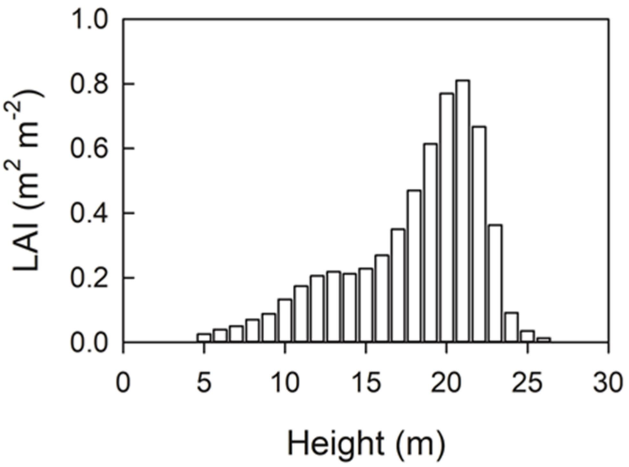

Supplementary Figure S4. LAI in each stratum with 1-m-intervals from the ground.

As a consequent, integrated canopy C gain for all strata in July, during a photosynthetically most vigorous period, was 24.9, 21.3, and 15.8 mol m^{-2} on the ground surface basis for optimal, even-E, and uniform leaf N distribution, respectively. Here, even-E N distribution showed a decrease in canopy C gain by 15% compared with optimal N distribution, while uniform N distribution showed a further decrease by 36%.

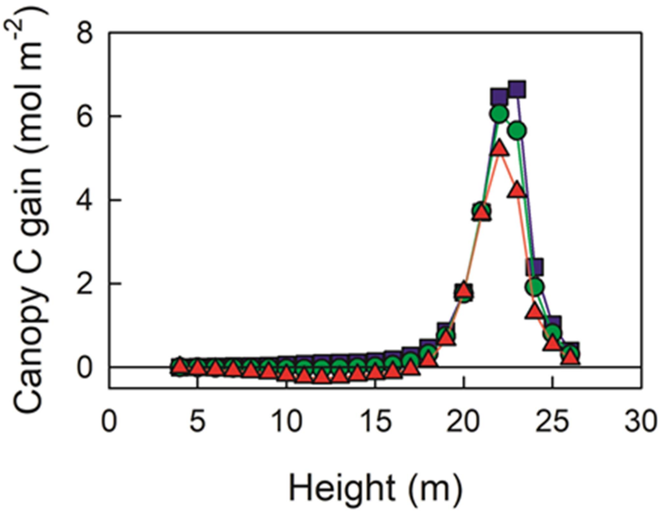

Supplementary Figure S5. Canopy C gain during July in each stratum with 1-m-intervals, with optimal (blue square), even-E (green circle), and uniform (red triangle) N distributions. Canopy C gain is based on the ground surface area.
